# Clinical characteristics and surgical treatment of ureteral endometriosis: our experience with 40 cases

**DOI:** 10.1186/s12905-021-01349-7

**Published:** 2021-05-17

**Authors:** Kunlin Yang, Sida Cheng, Yukun Cai, Jiankun Qiao, Yangyang Xu, Xinfei Li, Shengwei Xiong, Ye Lu, Aobing Mei, Xuesong Li, Liqun Zhou

**Affiliations:** 1 Department of Urology, Peking University First Hospital, No. 8 Xishiku St, Xicheng District, Beijing, 100034 China; 2 Institute of Urology, Peking University, No. 8 Xishiku St, Xicheng District, Beijing, 100034 China; 3 National Urological Cancer Center, No. 8 Xishiku St, Xicheng District, Beijing, 100034 China; 4 Department of Obstetrics and Gynecology, Peking University First Hospital, Beijing, China; 5 Department of Urology, The Second People’s Hospital of Guiyang, Guizhou, China

**Keywords:** Ureteralendometriosis, Ureteroureterostomy, Ureteroneocystostomy, Nephroureterectomy, Case report

## Abstract

**Background:**

To present the experience with the surgical management of ureteral endometriosis (UE) in our single center.

**Methods:**

To present the experience with the surgical management of ureteral endometriosis (UE) in our single center. A retrospective analysis of 40 patients with UE who presented with intraoperative surgical findings of endometriosis involving the ureter and pathology-proven UE was performed.

**Results:**

Forty patients (median age, 42.5 years) with histological evidence of UE were included. Six (15%) patients had a history of endometriosis. Twenty-one (52%) patients had urological symptoms, and 19 (48%) patients were asymptomatic. All patients had hydronephrosis. The mean glomerular filtration rate (GFR) of the ipsilateral kidney was significantly worse than that of the contralateral kidney (23.4 vs 54.9 ml/min; *P* < 0.001). Twelve (30%) patients were treated with ureteroureterostomy (11 open approaches and 1 robotic approach). Twenty-two (55%) patients underwent ureteroneocystostomy (17 open approaches, 4 laparoscopic approaches and 1 robotic approach). Five patients underwent nephroureterectomy. One patient refused aggressive surgery and received ureteroscopic biopsy and ureteral stent placement. Thirteen (33%) patients required gynecological operations. Three (8%) patients in the open group suffered from major surgical complications. Nine (24%) patients received postoperative endocrine therapy. Twenty-eight (70%) patients were followed up (median follow-up time, 71 months). Twenty-four patients received kidney-sparing surgeries. The success rate for these 24 patients was 21/24 (87.5%). The success rates of ureteroneocystostomy and ureteroureterostomy were 15/16 (93.8%) and 5/7 (71.4%), respectively.

**Conclusions:**

Although UE is rare, we should remain vigilant for the disease among female patients with silent hydronephrosis. Typically, a multidisciplinary surgical team is necessary. For patients with severe UE, segmental ureteral resection with ureteroureterostomy (UU) or ureteroneocystostomy may be a preferred choice.

## Background

Endometriosis is a common gynecologic disorder in women of childbearing age, with a prevalence of 10–20% among the general female population [1]. When the endometriosis involves the urinary tract, it is referred to as urinary tract endometriosis (UTE). The prevalence of UTE is difficult to determine because approximately 50% of women with endometriosis may be asymptomatic [2]. The literature reports that the incidence of UTE ranges from 0.3 to 12% among all women affected by endometriosis [3].

Ureteral endometriosis (UE) is a relatively rare situation and is the second most common type of UTE after bladder endometriosis [4]. UE is usually unilateral, and the distal ureter is the most commonly affected site. Symptoms related to UE are often nonspecific, and the clinical presentation is usually asymptomatic [5]. Typically, UE is diagnosed incidentally during a gynecologic follow-up or annual health examination. However, the late diagnosis and treatment of UE might lead to a silent loss of renal function. In a study reported by Jadoul P et al., the risk of loss of renal function among patients with UE was 11.5% [6, 7].

At present, there are no standard treatments or guidelines to definitively diagnose and treat UE. Generally, the goals of treatment for UE are the relief of ureteral obstruction and protection of renal function. Management depends on the site and extent of UE. The aim of this retrospective study is to summarize our experience with and provide more information on UE.

## Methods

We performed a search of our surgical and urological pathology databases from May 2004 to May 2020 for cases of UE. Only patients who were treated in our urological department and who had intraoperative surgical findings of endometriosis involving the ureter and pathology-proven ureteral endometriosis were included. Patients with no evidence of pathology-proven ureteral endometriosis were excluded. We collected the demographic data, characteristics, presenting symptoms, and surgical data of the patients. Patients were contacted to attempt follow-up.

Traditionally, ureteroneocystostomy and ureteroureterostomy (UU) are performed by an open approach. In recent years, some procedures have been performed by laparoscopic or robotic approaches in our center. We defined the criteria for success as the relief of symptoms and hydronephrosis. The presence of any unresolved hydronephrosis symptoms or deterioration of hydronephrosis was considered failure. Statistical analysis was performed with Microsoft® Excel® 2019 for Windows. Pairs of samples were compared using t tests. A *P* value < 0.05 was considered to be statistically significant.

## Results

Forty-two patients were found from the surgical database. Two patients without pathological examination data were excluded. A total of 40 patients with histologic evidence of UE were finally included. The median age was 42.5 (range, 27–72) years. Six (15%) patients had a history of endometriosis. One patient had received hormonal therapy before surgery, and one patient had a history of previous endometriosis surgery. Of the 40 included patients, 4 (10%) had dysmenorrhea, 12 (30%) had flank pain, 1 (3%) had abdominal pain, 3 (8%) had hematuria, 1 (3%) had frequent urination and 19 (48%) had no symptoms (Table 1). Twenty patients had left ureter involvement, and 20 patients had right ureter involvement. All patients had hydronephrosis. The glomerular filtration rate (GFR) data from 24 (60%) patients who had received renal dynamic scan examinations preoperatively were collected. The mean GFR of the ipsilateral kidney was 23.4 ml/min and that of the contralateral kidney was 54.9 ml/min (*P* < 0.001).

**Table 1:** Patients’ characteristics and preoperative findings

Total number of patients	40
The median age (years, range)	42.5 (27–72)
	No. Pts (%)
History of endometriosis	6 (15%)
Previous hormonal therapy	1 (3%)
Previous surgery for endometriosis	1 (3%)
History of abortion	4 (10%)
History of cesarean section	8 (20%)
History of ovarian cystectomy	5 (13%)
Concomitant myoma of uterus	8 (20%)
History of hysterectomy	2 (5%)
History of ectopic pregnancy	3 (8%)
Presenting symptoms	
Dysmenorrhea	4 (10%)
Flank pain	12 (30%)
Abdominal pain	1 (3%)
Hematuria	3 (8%)
Frequent urination	1 (3%)
Asymptomatic	19 (48%)
Ureteral involvement	
Left	20 (50%)
Right	20 (50%)
Mean GFR under renal dynamic scan (ml/min), no. Pts (%)	24 (60%)
Affected-side kidney (range)	23.4 (0–51)
Healthy-side kidney (range)	54.9 (39–77)

**Fig. 1:**
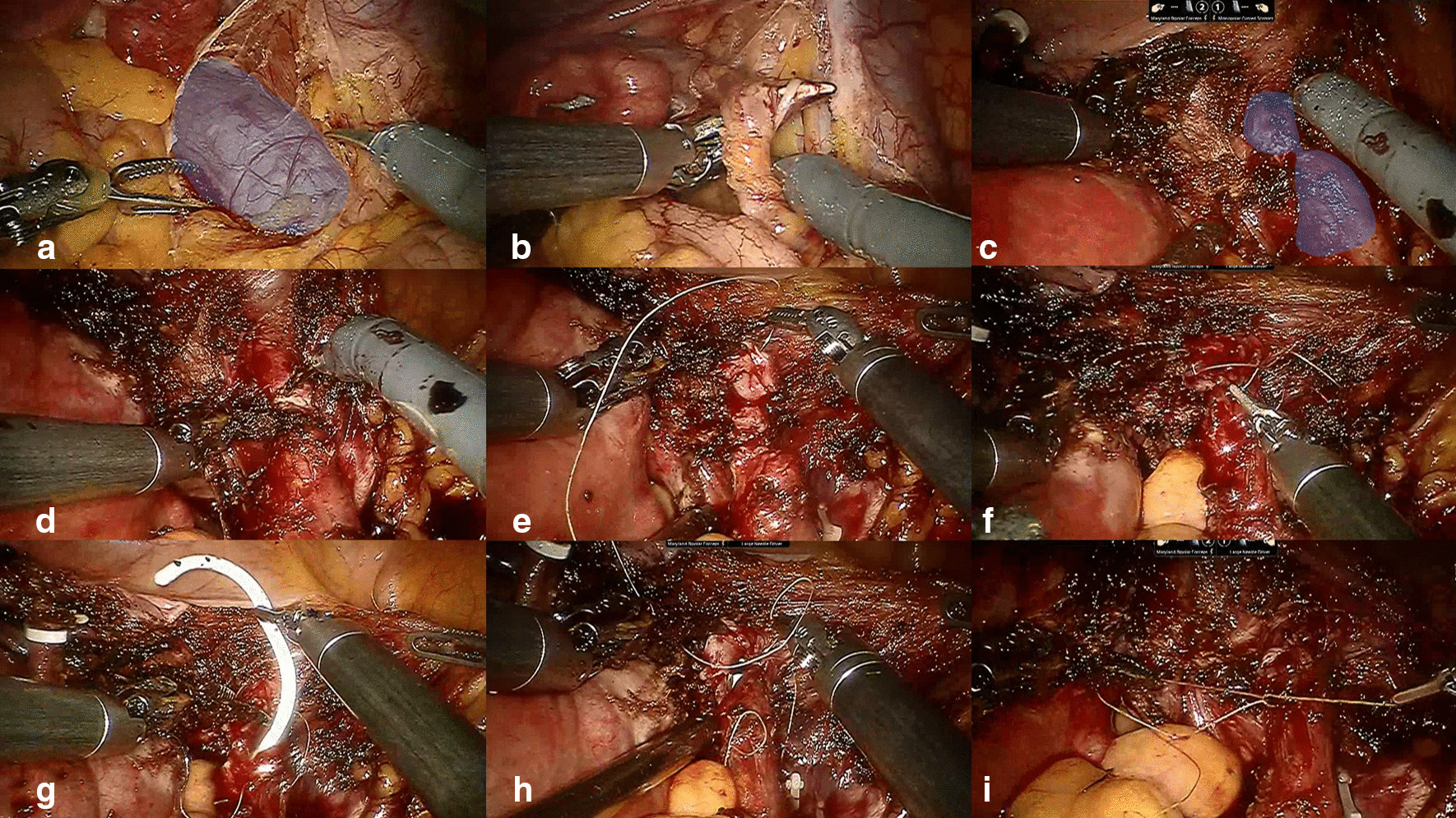
Robotic-assisted laparoscopic ureteroureterostomy. **a** Blue area shows the dilated ureter. **b** Cutting of the suspensory ligament of the right ovary and resection of the right ovary and endometrial lesion. **c** Dissection of the distal ureter presenting with stricture (blue area) close to the bladder. **d** Excision of the ureteral stricture. **e**–**i** Ureteroureterostomy

**Fig. 2:**
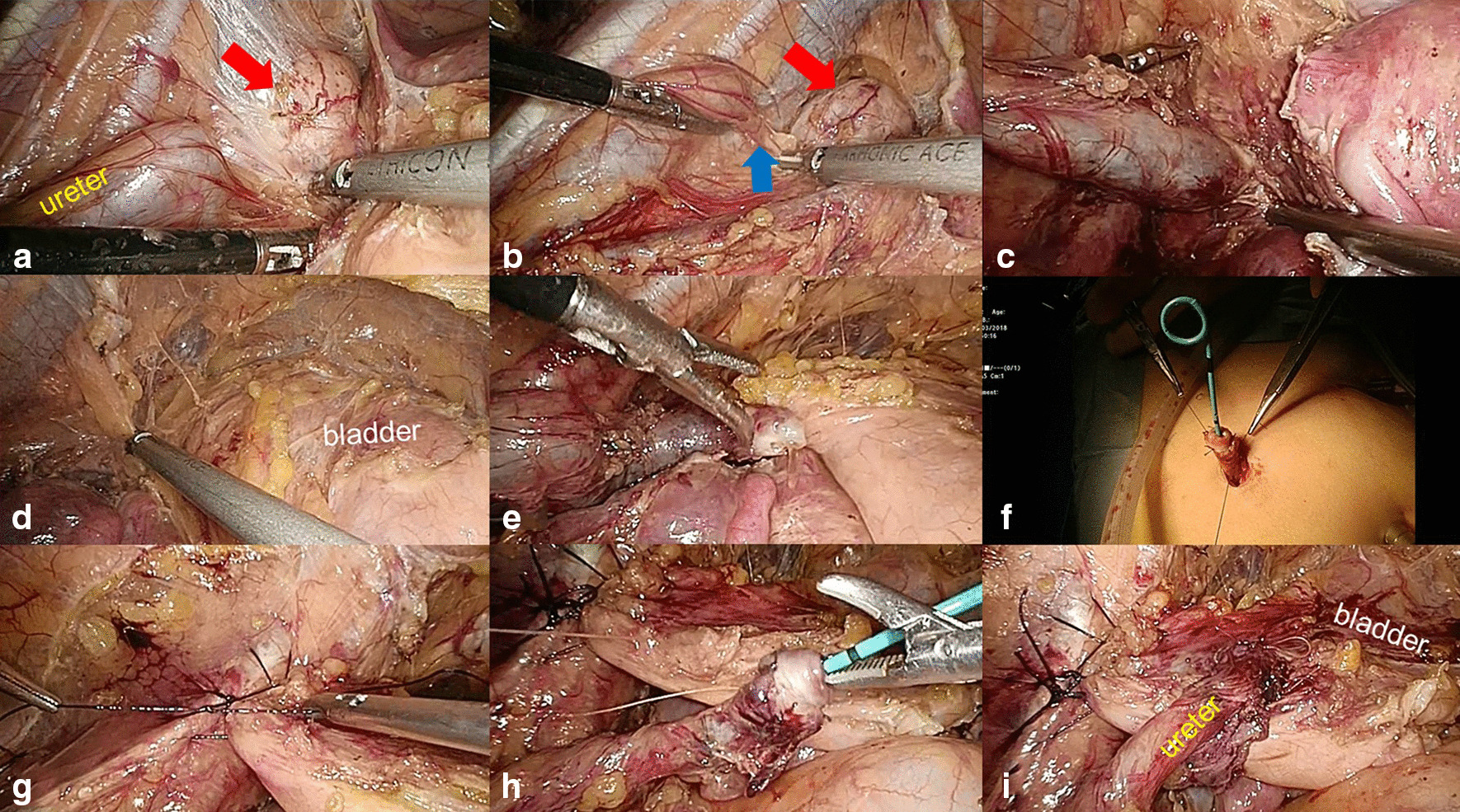
Laparoscopic ureteroneocystostomy with psoas hitch. **a** Dissection of the ureter and the endometrial nodule (red arrow). **b** The blue arrow shows the ureteral stricture, and the red arrow shows the endometrial nodule. **c** Excision of the endometrial lesion and cutting of the ureter. **d**, **e** Freeing of Retzius’ space. **f** Extracorporeal creation of a ureteral nipple. **g** Psoas hitching of the bladder. **h**, **i** Anastomosis of the ureter and the bladder

Twelve (30%) patients were treated with UU. Of these 12 patients, 11 underwent the open approach, and 1 underwent a robotic approach (Fig. 1). Of the 22 (55%) patients who underwent ureteroneocystostomy, 17 (43%) received open surgery, 4 (10%) received laparoscopic surgery and 1 (3%) received robotic surgery (Table 2). For some patients, ureteral reimplantation was performed with a psoas hitch (Fig. 2). Two patients were highly considered to have a ureteral tumor preoperatively, and 3 patients were diagnosed with a nonfunctioning ipsilateral kidney. Therefore, these 5 (13%) patients underwent nephroureterectomy. One patient refused to undergo aggressive surgery and finally received ureteroscopic biopsy and ureteral stent placement. There were 13 (33%) patients who required gynecological operations. The mean operative time was 152.4 min. The mean postoperative hospitalization was 6.7 days. Three (8%) patients in the open group separately suffered from major surgical complications (sigmoid colon injury/intestinal obstruction/blood loss requiring transfusion). Nine (24%) patients received postoperative endocrine therapy (Table 2).

**Table 2:** Intraoperative details and follow-up results

	No. pts (%)
Surgical procedures	
Ureteroureterostomy	12 (30%)
Open	11 (28%)
Robotic	1 (3%)
Ureteroneocystostomy	22 (55%)
Open	17 (43%)
Laparoscopic	4 (10%)
Robotic	1 (3%)
Nephroureterectomy	5 (13%)
Open	2 (5%)
Laparoscopic	3 (8%)
Ureteral stent placement	1 (3%)
Concomitant gynecologic operation	13 (33%)
Total hysterectomy + pelvic endometrial nodules resection	2 (5%)
Total hysterectomy + salpingo-oophorectomy	3 (8%)
Salpingo-oophorectomy + pelvic endometrial nodules resection	2 (5%)
Pelvic endometrial nodules resection	1 (3%)
Salpingo-oophorectomy	3 (8%)
Myomectomy	2 (5%)
Mean operative time, min (range)	152.4 (19–380)
Mean post-operative hospitalization, day (range)	6.7 (2–13)
Surgical complications, n	3 (8%)
Sigmoid colon injury/intestinal obstruction/blood transfusion	1/1/1
Postoperative endocrine therapy, n	9 (23%)
Total follow-up patients, n	28 (70%)
Ureteroureterostomy	7
Open	6
Robotic	1
Ureteroneocystostomy	16
Open	13
Laparoscopic	3
Nephroureterectomy	4
Open	1
Laparoscopic	3
Ureteral stent placement	1
Median follow-up time, month (range)	71 (11–150)
Positive follow-up results, n	3
Flank pain (open ureteroureterostomy group)	1
Recurrent urinary tract infection (open ureteroneocystostomy group)	1
Unresolved hydronephrosis (open ureteroureterostomy group)	1

Excluding 2 patients who had too recently undergone their procedures and 10 patients who could not be contacted, 28 (70%) patients were totally followed up. The median follow-up time was 71 months. Among these 28 patients, 7 underwent ureteroureterostomy (6 open surgeries and 1 robotic surgery), 16 underwent ureteroneocystostomy (13 open surgeries and 3 laparoscopic surgeries), 4 underwent nephroureterectomy (1 open surgery and 3 laparoscopic surgeries), and 1 underwent ureteral stent placement. Finally, we found that one patient (from the open ureteroneocystostomy group) had a recurrent urinary tract infection, and two patients (from the open ureteroureterostomy group) separately had persistent flank pain and unresolved hydronephrosis. Twenty-four patients received kidney-sparing surgeries, 21 of whom achieved the criteria for success for a success rate of 21/24 (87.5%). The separate success rates for ureteroneocystostomy and ureteroureterostomy were 15/16 (93.8%) and 5/7 (71.4%), respectively.

## Discussion

UE accounts for only 0.01% to 1.7% of endometriosis cases reported in the literature [8]. Typically, UE is very difficult to diagnose due to the absence of specific symptoms. Approximately half of the patients are diagnosed unexpectedly during routine health examinations. Our data show that 48% of patients were asymptomatic. For symptomatic women with UE, the three most common symptoms are severe dysmenorrhea (75%), dyspareunia (70%) and pelvic pain (60%) [4]. In our study, flank pain was the most common symptom. UE is usually unilateral. Some studies have reported that left UE is more severe than right UE [1, 4, 9]. The distal third of the ureter is the most frequently affected portion by endometriosis.

The pathogenesis of UE is still unknown; the most popular theory is retrograde menstruation [10]. However, this theory cannot completely explain isolated UE, in which no other endometrial implantation is found. Hydronephrosis is common when endometrial nodules are larger than 3 cm [11, 12]. UE is found predominantly among women with hydronephrosis and/or lesions larger than 4 cm [11]. UE lesions are very rarely isolated and are frequently associated with other kinds of endometriosis [13, 14]. Ureteral compression is common in patients with UE, particularly in patients with parametrial infiltration and a low body mass index [15].

Because physical examination often yields no positive findings for UE, rectovaginal palpation is necessary, which may provide a helpful indication of UE [13]. There are two types of UE: extrinsic and intrinsic. The extrinsic form involves compression of the ureteral wall and is more common than the intrinsic version, which involves invasion of the ureter and may originate from lymphatic or venous metastases [16]. When UE is suspected, all urologic causes of extrinsic and intrinsic ureteral stenosis should be considered, such as stones, primary megaureter, primary or secondary ureteral cancer, infection, retroperitoneal lymphadenopathy and idiopathic retroperitoneal fibrosis [6]. To differentiate these conditions, imaging techniques are needed [17]. Transvaginal and abdominal ultrasonography are the first-line examinations, as they can detect rectovaginal nodules at the distal third of the ureter and evaluate the degree of hydronephrosis and the thickness of the renal parenchyma [18]. However, the learning curve of transvaginal ultrasonography to identify bilateral ureter is deep and not all fellows can reach a level of competency by 50 cases examinations [19]. Magnetic resonance imaging (MRI) is highly accurate in detecting and predicting the type of UE. MRI is more sensitive but less specific than surgery in detecting intrinsic involvement, potentially overestimating the prevalence of intrinsic lesions [20]. Multislice computed tomography is an alternative to MRI, but it involves radiation, and the eventual enema can cause discomfort [6]. Renal scintigraphy should be performed when a decision between kidney preservation and nephrectomy is being considered.

When intrinsic UE needs to be distinguished from malignant ureteral tumors, a ureteroscopic biopsy may also be necessary to help make the final decision. However, as it is invasive and cannot detect extrinsic lesions, ureteroscopic biopsy is now rarely used in clinical practice [21].

Surgical treatment for UE aims to relieve ureteral obstruction and protect renal function. The main procedures include ureterolysis, ureterectomy with UU, ureteroneocystostomy and excision of all other endometrial lesions. In our hospital, most mild UE patients who only require ureterolysis are treated by gynecological doctors. If a more invasive procedure is needed, such as UU or ureteral reimplantation, the surgery is mainly performed by urologists. In this study, almost all cases were recommended from the gynecological department to the urological department. For some patients, the treatment process required a multidisciplinary team.

The choice of surgical procedure is determined by the severity and location of the lesions. Most lesions are located in the distal third of the ureter. Patients with less extensive endometriosis undergo ureterolysis and excision of all other lesions. Compared with ureteroneocystostomy, UU and ureterolysis have higher restenosis rates (11% and 8% versus 3%) [22]. Diego Raimondo et al. reported that near-infrared imaging with indocyanine green was a safe and useful tool to assess ureteral perfusion and guide surgical decision after conservative surgery [23]. For women with moderate to severe diseases, radical surgery is often required, including segmental resection with UU or ureteral reimplantation [24]. Lateral parametrial endometriosis is a condition that more aggressive surgery is required [25]. Paolo Donarini et al. reported that a UE patient treated by simple ureterolysis without ureteral resection was satisfied with the results at the 13-month follow-up [26]. Furthermore, nephrectomy or nephroureterectomy should be performed when renal function is less than 10–15% of baseline and the patient presents with symptoms such as flank pain, renovascular hypertension and recurrent urinary tract infection [27]. In our study, two patients considered to have ureteral cancer and three patients diagnosed with nonfunctional kidney disease ultimately underwent nephroureterectomy. Therefore, when the affected length of the ureter is more than 2 cm and UU cannot be performed tension free, ureteral reimplantation should be considered. Ureteral reimplantation changes the location of the distal end of the ureter from the bottom of the pelvic cavity to the lateral wall or the upper wall of the bladder, which we hypothesize may decrease the recurrence rate of ureteral stricture.

In the past ten years, open procedures have been the main type of surgery performed in our center. The development of laparoscopic and robotic techniques [28, 29] has led to their implementation in our center for performing some difficult operations. Especially for complex cases, robotic surgery has special advantages in performing anastomoses. As shown in Fig. 1, surgery was unlikely to be performed by open or laparoscopic approaches.

Among the patients described here, a 72-year-old woman who was initially suspected to have malignant ureteral cancer preoperatively was finally diagnosed with UE by postoperative pathology. Endometriosis tends to occur in women of childbearing age under 60 years. However, Haydon reported one of the oldest patients with endometriosis, aged 78 years [30]. This serves as a reminder that although rare, UE can also occur among elderly women.

Although instances of successful hormone therapy for UE have been reported [31, 32], medical treatment cannot address the fibrotic component of the lesion, which is mainly responsible for ureteral obstruction [33]. Therefore, UE with obvious ureteral obstruction should be treated surgically. For some patients with severe forms of the disease, postoperative adjuvant hormone therapy may be helpful [34, 35].

We must admit that there were some limitations in this study. This was a retrospective study with a relatively small sample that only included patients who were treated in our urological department. Despite the prevalence of minimally invasive surgery, most patients in this study still underwent open surgeries, which indicates that we should improve the surgical skills for minimally invasive UE surgery. A prospective and comparative study with a large sample size is needed for further study.

## Conclusions

Despite the rarity of UE, the difficulty in diagnosing the disease and the few guidelines for its surgical treatment, we should remain vigilant for UE among female patients with silent hydronephrosis. A multidisciplinary surgical team (which at least includes gynecologists and urologists) is necessary. For patients with severe ureteral involvement, segmental resection with UU or ureteral reimplantation may be the preferred choice.

## Data Availability

The data of the current study are available from the corresponding author upon reasonable request.

## Abbreviations

UTE: Urinary tract endometriosis
UE: Ureteral endometriosis
MRI: Magnetic resonance imaging
UU: Ureteroureterostomy
